# Role of TRP Channels in Shaping the Gut Microbiome

**DOI:** 10.3390/pathogens9090753

**Published:** 2020-09-16

**Authors:** Ravinder Nagpal, Santosh Kumar Mishra, Gagan Deep, Hariom Yadav

**Affiliations:** 1 Department of Internal Medicine-Molecular Medicine, Wake Forest School of Medicine, Winston-Salem, NC 27101, USA; rnagpal@wakehealth.edu; 2 Department of Molecular Biomedical Sciences, NC State Veterinary Medicine, Raleigh, NC 27606, USA; skmishra@ncsu.edu; 3 Department of Cancer Biology, Wake Forest School of Medicine, Winston-Salem, NC 27157, USA; gdeep@wakehealth.edu; 4 Department of Microbiology and Immunology, Wake Forest School of Medicine, Winston-Salem, NC 27101, USA

**Keywords:** intestinal microflora, microbiota, pain, transient receptor potential, TRP channels, TRPA1, TRPV1

## Abstract

Transient receptor potential (TRP) channel family proteins are sensors for pain, which sense a variety of thermal and noxious chemicals. Sensory neurons innervating the gut abundantly express TRPA1 and TRPV1 channels and are in close proximity of gut microbes. Emerging evidence indicates a bi-directional gut–brain cross-talk in several entero-neuronal pathologies; however, the direct evidence of TRP channels interacting with gut microbial populations is lacking. Herein, we examine whether and how the knockout (KO) of TRPA1 and TRPV1 channels individually or combined TRPA1/V1 double-knockout (dKO) impacts the gut microbiome in mice. We detect distinct microbiome clusters among the three KO mouse models versus wild-type (WT) mice. All three TRP-KO models have reduced microbial diversity, harbor higher abundance of Bacteroidetes, and a reduced proportion of *Firmicutes*. Specifically distinct arrays in the KO models are determined mainly by *S24-7*, *Bacteroidaceae*, *Clostridiales*, *Prevotellaceae, Helicobacteriaceae, Rikenellaceae,* and *Ruminococcaceae*. A1KO mice have lower *Prevotella, Desulfovibrio, Bacteroides*, *Helicobacter* and higher *Rikenellaceae* and *Tenericutes*; V1KO mice demonstrate higher *Ruminococcaceae, Lachnospiraceae, Ruminococcus, Desulfovibrio* and *Mucispirillum*; and A1V1dKO mice exhibit higher *Bacteroidetes, Bacteroides* and *S24-7* and lower *Firmicutes, Ruminococcaceae, Oscillospira, Lactobacillus* and *Sutterella* abundance. Furthermore, the abundance of taxa involved in biosynthesis of lipids and primary and secondary bile acids is higher while that of fatty acid biosynthesis-associated taxa is lower in all KO groups. To our knowledge, this is the first study demonstrating distinct gut microbiome signatures in TRPA1, V1 and dKO models and should facilitate prospective studies exploring novel diagnostic/ therapeutic modalities regarding the pathophysiology of TRP channel proteins.

## 1. Introduction

The transient receptor potential (TRP) channel family of proteins are known as a detector for various external and internal stimuli and react to an array of changes in temperatures, acidic and basic pH, osmolarity, odorants, chemicals, intracellular lipid mediators such as anandamide and lipoxygenase products, fatty acids and mechanical stimuli [1,2,3]. Among these, temperature sensation through TRP channels provides critical information about environment and triggers perceptual reflexes and responses ranging from sensation to pain. Hence, TRP receptors that sense thermal- and chemical-induced pain are an important receptor family involved in generation of pain. Expressed by primary afferent neurons, both TRPA1 (activated by extreme cold) and TRPV1 (major mammalian sensor of noxious heat) function as a sensor for detecting inside and outside painful stimuli, temperature and inflammation [4]. Sensory neurons innervating the gut abundantly express TRPA1 and TRPV1 channels [5], and are in close proximity to a highly diverse and complex community comprising trillions of microbes living in the intestinal tract (gut microbiome).

Recent and ever-mounting evidence has revealed the critical role of the gut microbiome in a wide array of pathologies including, but not limited to, gastrointestinal, metabolic, cardiovascular, neurological, and psychiatric disorders [6]. However, data on the possible role of the gut microbiome in pain-related pathophysiology outside of the gastrointestinal tract are scarce. Given the rapidly emerging evidence related to the interactions between the gut microbiome and the central nervous system (CNS), also known as the ‘gut–brain axis’ [7], it is reasonable to hypothesize that the gut microbiome may also be linked to the physiology of pain modulation through the prominent pain receptors including TRPs [8,9,10]. Several animal studies have shown that the gut microbiome plays an important role in the development of visceral pain [11,12] as well as neuropathic pain [13]. Several human studies have also reported gut microbiome alterations in patients with several visceral pain disorders including irritable bowel syndrome (IBS) [14,15,16], chronic dysfunctional pelvic pain [17,18], chronic fatigue syndrome [19,20], rheumatoid arthritis and spondyloarthropathies [21,22], and fibromyalgia [23,24]. Despite these reports, there is still no evidence of such gut microbiome alterations in the milieus involving non-visceral pain. Further, the specific and precise interaction between gut microbiome and TRP channels remains unclear. Emerging evidence indicates that the communication between gut and brain is bidirectional [7]. Neuronal lines of communication help the brain to regulate and control the gastrointestinal processes, while signals relayed back by the gastrointestinal tract (GIT) can influence both perception and host behavior [7]. The gut microbiome and its metabolites can influence the gut–brain axis [25] to change sensation in peripheral nerves, and perception circuits in the brain. Similarly, brain and sensory neurons innervating the gut can also influence microbial communities; however, whether and how TRP channels can influence the gut microbiome remains unknown.

Most recently, the involvement of bioactive lipids, such as the N-acylethanolamine (NAE) family whose main members are N-arachidonoylethanolamine (AEA), palmitoylethanolamide (PEA) and oleoilethanolamide (OEA), as well as the short-chain fatty acids (SCFAs) such as butyrate in the gut have been found to modulate peripheral and central neuronal processes [26]. More than 50 arachidonic acid- and linoleic acid-metabolites as well as lysophospholipids, and isoprenoids are among the endogenous TRP-channel sensitizers, activators and inhibitors, and modulate neuronal sensations [27,28,29,30,31]. Therefore, the lipid metabolism modulation in the gastrointestinal tract and the gut microbiome can influence the neuronal sensation and vice-versa. However, direct evidence of gut microbiome regulation by TRP channels is still lacking. Herein, we for the first time examine whether and how the individual knockout of TRPA1 and TRPV1 channels as well as the combined TRPA1/V1 double-knockout in mice impacts the gut microbiome and its metabolic pathways.

## 2. Results

### 2.1. Deletion of TRP-A1 and -V1 Channels Individually (KO) or Both -A1/V1 Together (dKO) Distinctly Impacts Gut Microbiome Diversity

**Figure 1:**
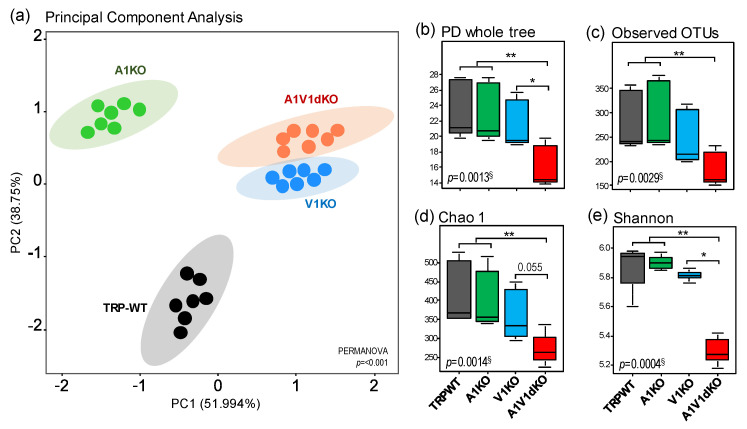
Distinct patterns of gut microbiome diversity in mouse models of transient receptor potential (TRP)A1 knockout, TRPV1 knockout and TRPA1V1 double-knockout as compared to each other as well as to the TRP wild-type counterparts. (**a**) Beta-diversity (PCoA) plot of the gut microbiome in mouse models of TRPA1 knockout (A1KO), TRPV1 knockout (V1KO) and TRPA1V1 double-knockout (A1V1dKO) versus TRP wild-type (TRP-WT) counterparts. (**b–e**) Alpha-diversity indices i.e., PD whole tree (phylogenetic diversity), Observed number of operational taxonomic units (OTUs), Chao1 (species richness) and Shannon (species evenness) in mouse models of TRPA1 knockout (A1KO), TRPV1 knockout (V1KO) and TRPA1V1 double-knockout (A1V1dKO) versus TRP wild-type (TRP-WT) counterparts. ^§^ Kruskal–Wallis test; * *p* < 0.05, ** *p* < 0.001 (pair-wise Dunn’s post-hoc test).

The analysis of β-diversity (a measure of microbial diversity differences among the groups) of the gut microbiome reveals that the three different mouse models of TRPA1, TRPV1 and TRPA1/V1 dKO harbor distinct signatures of the gut microbiome when compared to their age- and gender-matched wild-type (TRP-WT) counterparts (Figure 1a). The β-diversity of microbiome signatures of three KOs are clustered distinctly from each other as well as from WT counterparts. The microbiomes of V1 KO and A1/V1 dKO are clustered relatively close to each other while the A1 KO is relatively closer to WT but still clearly distinct from other groups (Figure 1a, Supplementary Figure S1). Further analyses of α-diversity (a measure of microbial diversity within samples) indices (i.e., Chao1 (species richness), number of operational taxonomic units (OTUs)detected, phylogenetic diversity (PD) and Shannon index (species evenness)) also show that the three KOs mice harbor distinct populations of gut microbes indicated by significant differences in the α-diversity indices of the gut microbiome. The A1/V1 dKO mice show the lowest and most distinct pattern in terms of all α-diversity indices (Figure 1b–e). Overall, the α-diversity in A1 KO mice remains similar to that in WT mice while the V1 KO mice show marginally reduced indices of PD whole tree, observed number of OTUs, and species richness (Chao1) and evenness (Shannon) when compared to WT and A1 KO counterparts (Figure 1b–e). In contrast, A1/V1 dKO mice demonstrate remarkably reduced phylogenetic diversity, observed number of OTUs and species richness and evenness as compared to all of the other three groups of mice (Figure 1b–e).

### 2.2. Deletion of TRP-A1 and -V1 Channels Individually or Both -A1/V1 Together Generates Distinct Microbiome Composition in the Mouse Gut

**Figure 2:**
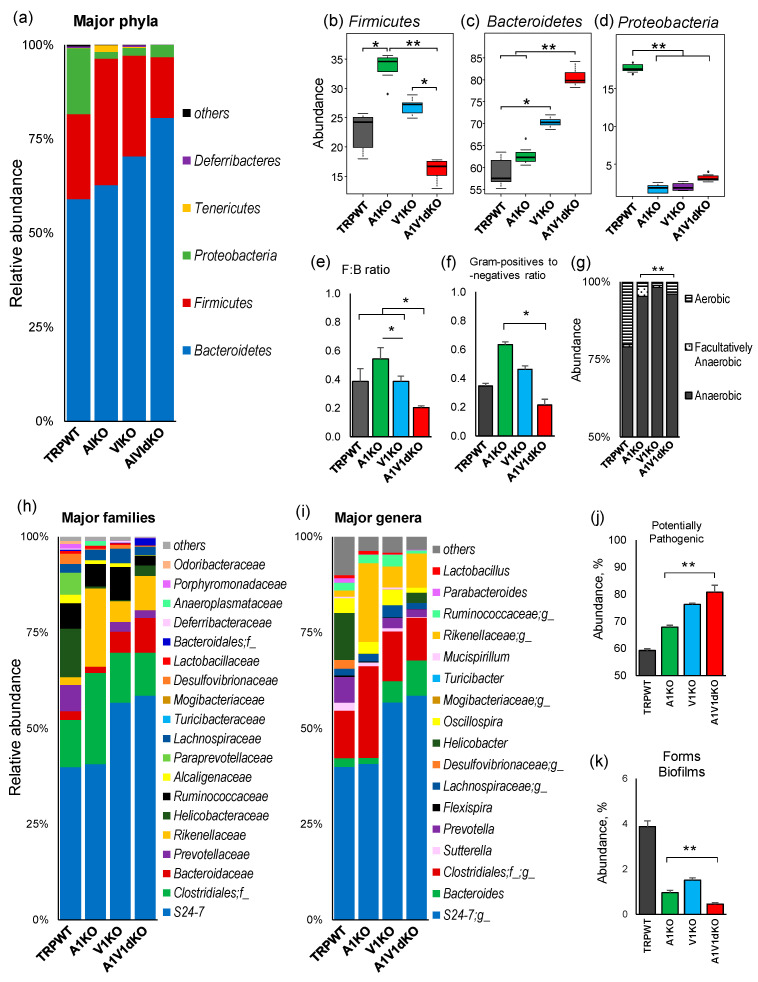
Gut microbiome composition differs in mouse models of TRPA1 knockout, TRPV1 knockout and TRPA1/V1 double-knockout as compared to each other as well as to their TRP wild-type counterparts. (**a**) Gut microbiome composition at bacterial phylum level; (**b**–**d**) the abundance of major phyla; (**e**) ratio of Firmicutes to Bacteroides and (**f**) ratio of Gram-positive to Gram-negative taxa; (**g**) ratio of anaerobic to aerobic bacteria; and (**h**,**i**) microbiome composition at the level of major bacterial families (**h**) and genera (**i**); and (**j**,**k**) proportion of potentially pathogenic bacteria (**j**) and biofilm forming bacteria (**k**), in the mouse models of TRPA1 KO, TRPV1 KO and TRPA1/V1 dKO versus TRP-WT counterparts. * *p* < 0.05, ** *p* < 0.001.

The relative abundance of major phyla is found to be significantly distinct in three KO groups as compared to WT counterparts as well as to each other (Figure 2a), suggesting that each of these three TRP-genotypes developed a unique microbial phyla signature. All three KO groups have an increased proportion of phylum *Bacteroidetes* with the proportion being significantly highest in A1/V1 dKO mice followed by V1KO while the proportion in A1KO mice is only marginally higher (Figure 2a,c). Oppositely, the abundance of phylum *Firmicutes* demonstrates an inverse (of phylum *Bacteroidetes*) pattern characterized by the lowest proportion in the dKO mice versus all of the other groups (Figure 2a,b). Overall, the A1 KO mice show the highest ratio of *Firmicutes* to *Bacteroidetes* while the ratio in V1 KO mice is equivalent to that in WT mice, in contrast to the A1/V1 dKO mice that demonstrate a significantly lower ratio compared to all other three groups of mice (Figure 2e). Furthermore, the abundance of the third major phylum i.e., *Proteobacteria* is remarkably diminished in all of the three KO groups compared to WT counterparts (Figure 2d). The overall ratio of Gram-positive and -negative bacteria is significantly higher in A1- and V1-KO but significantly lower in A1V1dKO mice compared to WT counterparts (Figure 2f). In addition, all of the three KO models harbor remarkably higher ratios of obligate anaerobic bacteria over aerobic bacteria (Figure 2g). Subsequent analysis of relative abundance at the level of bacterial families and genera also reveal specifically distinct and unique arrays in all the four groups wherein the differences in the KOs versus WT groups are determined largely by the members of the families *S24-7*, *Bacteroidaceae*, unclassified *Clostridiales* family, *Prevotellaceae*, *Helicobacteriaceae*, *Rikenellaceae*, and *Ruminococcaceae* (Figure 2h–i). The organism-level phenotype analysis reveals a significantly higher proportion of OTUs corresponding to potential pathogenic bacteria in all the three KO models, with the proportion being highest in A1V1dKO followed by V1KO and A1KO (Figure 2j). Furthermore, the proportion of potential biofilm forming bacteria is significantly lower in all the three KO models versus WT mice (Figure 2k). In addition, the proportion of bacteria containing mobile elements is significantly lower while that of stress tolerant bacteria is higher in all the three KO groups versus WT counterparts (Supplementary Figure S2).

**Figure 3:**
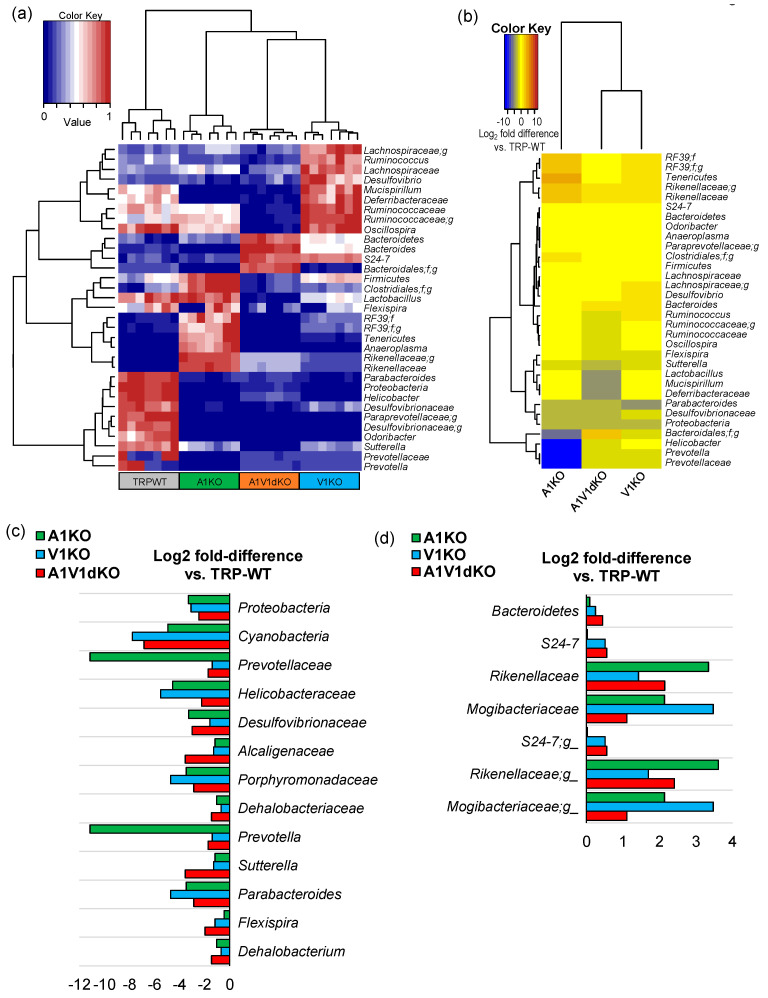
Differences in the abundance of various gut bacteria in mouse models of TRPA1 knockout, TRPV1 knockout and TRPA1V1 double-knockout as compared to each other as well as to the TRP wild-type counterparts. (**a,b**) Hierarchical clustering heap-map depicting distinct arrays characterized by different abundance levels (**a**) and Log_2_ fold-differences (**b**) of major gut bacterial phyla, families and genera in mouse models of TRPA1 KO, TRPV1 KO and TRPA1/V1 dKO versus TRP-WT counterparts. (**c,d**) Major gut bacterial phyla, families and genera that were found to be reduced (**c**) or increased (**d**) in all of the three mouse models i.e., A1 KO, V1KO and A1V1dKO versus TRP-WT counterparts.

The analysis of relative abundance of major (top 15) bacterial taxa by hierarchal clustering clearly assorts the whole cohort into four distinct clusters driven by the type of the KO (Figure 3a), wherein A1 KO clusters close to the WT group whereas the V1 KO and A1/V1dKO groups are clustered as separate distinct clusters. Similar clustering is demonstrated by the further analysis of Log_2_-fold difference in the relative abundance of these bacterial taxa in KO versus WT mice wherein V1 KO and A1/V1 dKO are clustered together and distinctly apart from A1 KO group (Figure 3b). The A1 KO cluster is characterized mainly by the markedly lower abundance of *Prevotella, Desulfovibrio, Bacteroides,* and *Helicobacter* and a higher proportion of *Rikenellaceae* and *Tenericutes*; V1 KO mice demonstrate a relatively higher abundance of *Ruminococcaceae, Lachnospiraceae, Ruminococcus, Desulfovibrio*, and *Mucispirillum*, whereas the A1/V1 dKO groups are characterized by a higher proportion of members belonging to the taxa *Bacteroidetes, Bacteroides* and *S24-7* and a lower abundance of *Firmicutes, Ruminococcaceae, Oscillospira, Lactobacillus* and *Sutterella* (Figure 3b). Further analysis of major bacterial taxa reveals several bacteria that are lower (Figure 3c) or higher (Figure 3d) in all the three knockout groups (although at different magnitudes) compared to the WT mice. Bacteria belonging to phyla *Proteobacteria* and *Cyanobacteria*, and families *Prevotellaceae*, *Helicobacteriaceae*, *Porphyromonadaceae* and *Desulfovibrionaceae* are lower in all the KOs mice whereas the population of phylum *Bacteroidetes*, and families *S24-7*, *Rikenellaceae* and *Mogibacteriaceae* is increased in all the three KOs compared to WT mice (Figure 3c,d).

### 2.3. Mice with TRP-A1 KO, -V1 KO and -A1/V1 dKO Genotypes Present Unique Gut Microbiome Signatures

**Figure 4:**
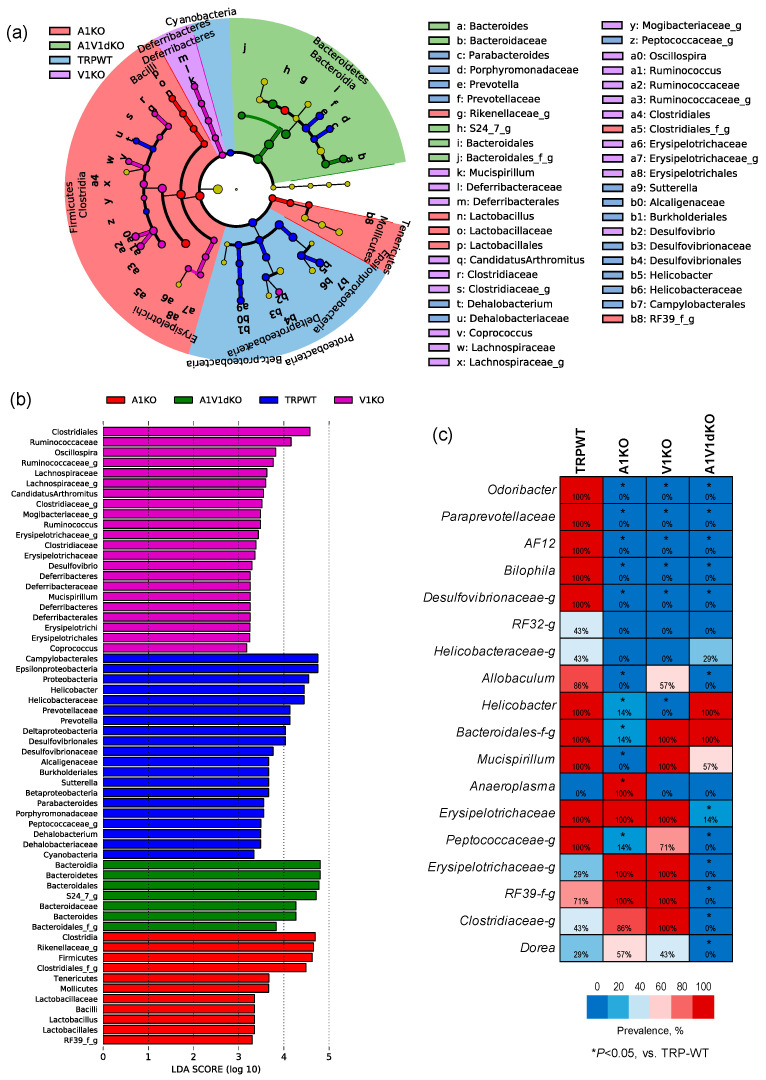
Unique gut microbiome signatures associated with specific mouse models of TRPA1 knockout, TRPV1 knockout and TRPA1V1 double-knockout. Linear Discriminatory Analysis (LDA) Effect Size (LEfSe) plot (**a**) and cladogram (**b**) showing bacterial taxa that are unique in mouse models of TRPA1 KO, TRPV1 KO and TRPA1/V1 dKO as well as in TRP-WT counterparts. (**c**) Differences in the detection rates of major bacterial phyla, families and genera in mouse models of TRPA1 KO, TRPV1 KO and TRPA1/V1 dKO versus TRP-WT counterparts.

We then perform Linear discriminatory analysis (LDA) Effect Size (LEfSe), analysis to identify bacterial taxa that are unique in each group (Figure 4a,b). As demonstrated by the LEfSe- generated cladogram (Figure 4a) as well as in terms of the LDA score (Figure 4b), the A1KO mice harbor a higher proportion of *Firmicutes, Clostridia, Rikenella, Mollicutes* and *Lactobacillus* whereas V1KO mice microbiomes are enriched with members of *Clostridiales, Ruminococci, Lachnospira, Mogibacteria, Deferribacteriaceae*, and *Erysipelotrichaceae*. The A1V1dKO group is characterized by a higher proportion of the members of the phylum *Bacteroidetes* including the representative genus *Bacteroides* as well as the next representative family *S24_7*. Furthermore, all the three knockout groups have lower populations of *Proteobacteria, Helicobacter, Prevotella, Sutteralla, Parabacteroides, Dehalobacterium* and *Cyanobacteria* when compared to the WT mice.

In addition to these uniquely abundant bacterial signatures, we also find several taxa that are unique in terms of their detection rate in the feces of mice with different TRP genotypes (Figure 4c). As shown in the form of a heat-map in Figure 4c, OTUs belonging to the bacterial taxa *Odoribacter, Paraprevotella, AF12, Bilophila* and *Desulfovibrionaceae* are detected in all the WT mice (detection rate: 100%) but remain undetected (detection rate: 0%) in all of the three KO mice, thereby indicating their association with the host TRP-genotype status. In addition, OTUs belonging to taxa *Peptococcaceae, Erysipelotrichaceae, RF39, Clostridiaceae* and genus *Dorea* are vanished in A1/V1 dKO mice but not in -A1 or -V1 KO mice. The genus *Anaeroplasma* is detected in A1 KO mice but remains completely undetected in WT as well as in V1 and A1/V1 dKO mice. In addition, the detection rate of *Mucispirillum* and an unclassified family of *Bacteroidales* is significantly lower only in A1 KO mice but not in V1 KO or A1/V1 dKO mice. On the other hand, the genus *Allobacullum* remains undetected in A1 KO and A1/V1 dKO mice but is detected in 57% of V1 KO mice. This indicates that TRP-gene deletion not only changes the overall microbiome signature, but also represents a unique microbial signature for each genotype.

### 2.4. Microbiome-Related Metabolic Functions Involved in Biosynthesis and Metabolism of Lipid and Fatty Acids are Distinctly Modulated upon Deletion of Specific TRP Channels

**Figure 5:**
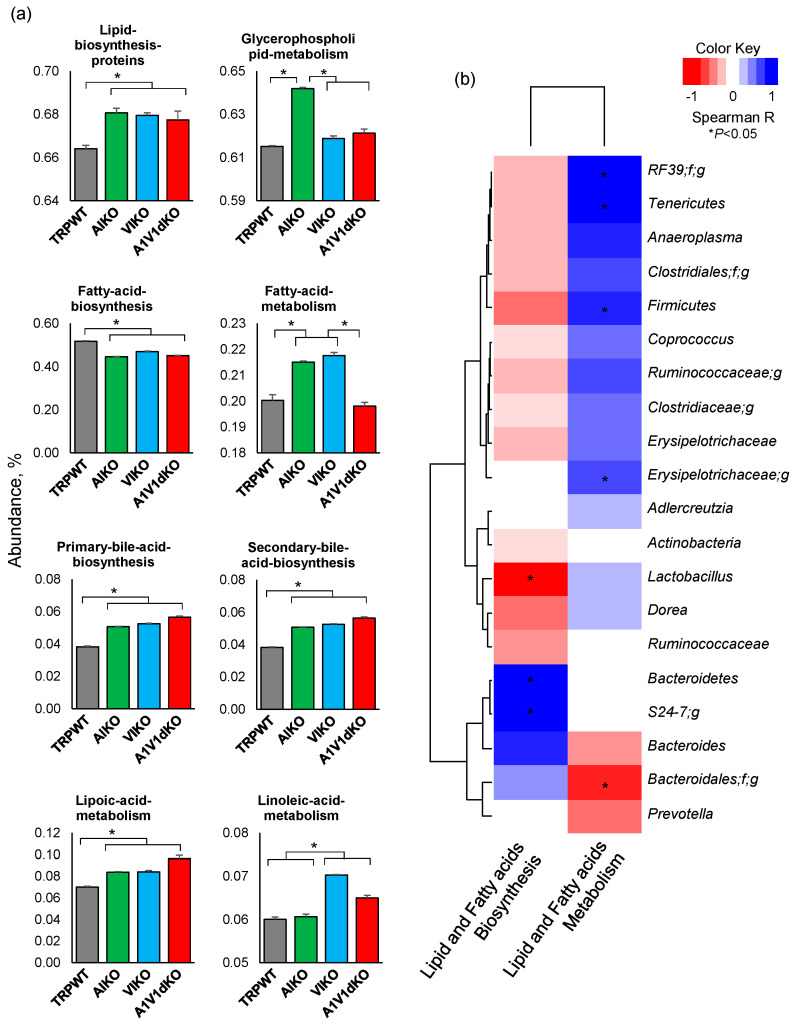
Differences in the functional analysis of the gut microbiome in mouse models of TRPA1 knockout, TRPV1 knockout and TRPA1V1 double-knockout. (**a**) The abundance of the predicted gut microbial metagenomic functions related to the lipids and fatty acids biosynthesis and metabolism pathways (Level 3 Kyoto encyclopedia of genes and genomes (KEGG) pathway) in mouse models of TRPA1 KO, TRPV1 KO and TRPA1/V1 dKO as well as in TRP-WT counterparts. (**b**) The hierarchical heat-map depicting the correlation of specific gut bacterial phyla, families and genera with functional metagenomic KEGG pathways related to the biosynthesis and metabolism of lipids and fatty acids.

The analysis of Phylogenetic Investigation of Communities by Reconstruction of Unobserved States (PICRUSt)-inferred functional categorization of the gut microbiome followed by the Clusters of Orthologous Groups (COG) classification revealed many metagenomic functions related to the biosynthesis or metabolism of various lipids, fatty acids, carbohydrates, amino acids, vitamins, and other co-factors that were found to be significantly different between the different host genotypes. Of all these unique predicted functions (Supplementary Figure S3), the abundance of Kyoto encyclopedia of genes and genomes (KEGG) pathways particularly associated with the biosynthesis or metabolism of lipid and fatty acids were found to be unique in each of the three KO mouse models (Figure 5a). Interestingly, the abundance of gene families associated with the biosynthesis of lipids as well as the primary and secondary bile acids was found to be significantly higher in all of the three KO mice versus the WT mice. In contrast, the abundance of OTUs associated with overall fatty acid biosynthesis was found to be significantly lower in all these three TRP-KO groups. Whereas, the abundance of fatty acid metabolism-related families was significantly higher only in A1- and V1- KOs but not in dKO mice. The abundance of glycerophospholipid metabolism related OTUs was significantly higher in A1 KO mice as compared to WT as well as to V1 KO and TRPA1/V1 dKO mice. In addition, the abundance of OTUs associated with linoleic acid metabolism was significantly higher in V1 KO and -A1/V1dKO but not in TRPA1 KO mice. Further analysis of the direct correlation of the abundance of these functional orthologs with the abundance of bacterial taxa revealed a distinct array of correlations of specific bacteria with the biosynthesis or metabolism of lipids and fatty acids (Figure 5b). OTUs belonging to the taxa *Enterococcaceae-RF39, Tenericutes, Anaeroplasma, Clostridiales, Firmicutes, Coprococcus, Ruminococcaceae, Erysipelotrichaeae, Aldercreutzia, Lactobacillus,* and *Dorea* were correlated negatively with the metagenomic functions related to lipids and fatty acids metabolism but positively with those related to biosynthesis. In contrast, OTUs belonging to bacterial taxa *Bacteroidetes, S24_7, Bacteroides* and *Prevotella* correlated positively with biosynthesis but negatively with the metabolism of lipids and fatty acids (Figure 5b). The data indicate that TRPA1, TRPV1 and TRPA1/V1 deletion developed unique microbiome signatures that perform unique metabolic functions for modulation of lipid and bile acid metabolisms.

## 3. Discussion

In this study, we examine the gut microbiome diversity and composition in TRPA1, V1, and A1/V1 knockout mice in comparison to their wild-type counterparts and identify distinct sets of gut bacterial signatures associated with the knockout of specific TRP genes involved in the pathophysiology of pain.

The gut microbiome analysis clearly reveals four distinct clusters (Figure 1a) specific for four separate groups of mice viz. TRPA1 KO, -V1 KO, -A1/V1 dKO and TRP wild-type mice, thereby indicating different microbiome signatures between these four groups of mice. In addition, the α-diversity indices demonstrate a patterned decline in the bacterial diversity in the KO versus WT groups (Figure 1b) with diversity being modestly reduced in A1KO followed by considerably reduced in V1 KO while being remarkably reduced in the A1/V1dKO mice. Interestingly, both α- and β-diversity analyses show V1 KO and A1/V1 dKO mice to be closer to each other in comparison to TRP-WT and A1KO, indicating that both A1 and V1 knockouts may induce very distinct magnitudes of impact on the gut microbiota diversity and composition. Notably, this decline in the microbial diversity concurs with several studies reporting reduced microbiome diversity in several pain-related disorders, such as fibromyalgia and myalgic encephalomyelitis, chronic fatigue syndrome [19,23,24]. Interestingly, our further analyses in KO groups demonstrate a reduction in the abundance of several bacterial taxa typically associated, as commensals, with a typical murine microbiome, such as *Prevotella, Helicobacter, Desulfovibrio, Sutterella, Parabacteroides,* and *Dehalobacterium* (Figure 3c). These differences can be seen even at the highest level of taxonomic classification i.e., at the phylum level which shows a clear significant reduction in *Proteobacteria* in unison with an expansion in the abundance of *Bacteroidetes* (Figure 2a–d), thereby suggesting a dysbiotic (abnormal) microbiome spectrum in TRP KO versus WT mice. Since there is no consensus as such at present on the use of the term “dysbiosis” or its meaning, it may be noted that the term “dysbiosis” in here is simply used to refer to a different gut microbiome composition in experimental groups versus wild-type (without any inference to whether it is casually or causally associated with the genetic manipulation or a disease manifestation). Besides the evoked noxious painful response (and the microbiome differences reported in here), these mice do not display any other major phenotypes.

Dysbiosis of the gut microbiome is also commonly reported to be associated with an abnormal intestinal epithelial permeability (‘leaky gut’) which may abnormally increase the interaction of gut bacteria with the host intestinal immune system (e.g., gut-associated lymphoid tissues) as well as the enteric nervous system (enteric neurons, neurotransmitters, etc.) eventually leading to increased episodes of local inflammation [32]. Such dysbiotic events have been previously reported in patients with chronic abdominal pain and also in patients with chronic widespread pain such as fibromyalgia [23,24]. In our TRP KO models, we find a decrease in the abundance of several members of the *Prevotellaceae, Paraprevotellaceae, Desulfovibrionaceae, Helicobacteriaceae* and *Clostridiaceae* and an increase in the proportion of *Bacteroidetes, Rikenellaceae* and *Mogibacteriaceae*. Such differences in the intestinal carriage of gut commensal along with a reduced overall diversity of bacteria, many of which are gut commensals (as seen in the wild-type counterparts) and are also involved in the nutrient digestion and the production of beneficial SCFAs, might also hint that such spectrum of gut dysbiosis might also be implicated in the comorbidities associated with the pathophysiology of pain. Hence, further studies undertaking a more inclusive and particularly longitudinal investigation (the lack of which is a limitation of our small study) are warranted to corroborate these findings as well as to gain deeper understanding of this otherwise under-explored microbiome–pain link.

The role of lipids and fatty acids in the propagation of pain is known to be like two opposite sides of the same coin, which can either increase the pain sensitivity by activating the nociceptors present on the sensory neurons [33,34,35,36] or attenuate pain which is mediated by interaction with TRP channels [37,38,39]. Lipid mediators have previously been demonstrated to increase the pain sensitivity by activating G-protein coupled receptors (GPCR) that are linked through TRP channels acting downstream of the GPCR [40]. The anti-nociceptive effect of resolvins, the specialized proresolving lipid mediators, has been demonstrated to be due to the resolution of inflammation, which in turn facilitates reduction in pain sensitivity [41]. In addition, resolvins are the first endogenous inhibitors of TRPA1 and TRPV1 receptors that reduce pain by shutting the activity of these receptors by a G-protein regulated mechanism [42]. During inflammation, the levels of resolvins are increased significantly in our body, although the potential mechanism(s) involved in this significant increase remains unknown. Despite the fact that these lipid mediators have two opposite effects on pain, it might be plausible that what regulates their synthesis and metabolism might be potentially associated with the gut microbiome. TRP channels act as an internal and external sensor or gate-keeper to regulate the gut microbiome, which in turn modulate pain sensitivity by regulating its synthesis and metabolism. Most importantly, our data on changes in gut bacteria that are involved in lipid biosynthesis and metabolism in TRP-deficient mice will provide some important clues about the role of these microbiota in nociceptive and anti-nociceptive processing. Taken together, the exact role of these gut microbial groups needs further exploration.

TRP channels are expressed all along the gastrointestinal system wherein they act as molecular sensors and transducers and play a role in the regulation of a variety of functions [43,44]. Studies have shown that the TRP channels are expressed by primary afferent sensory neurons that arise from ganglia and enteric neurons which, conjunctly, innervate the intestinal tract [45,46]. Emerging evidence also shows the presence of TRP channels in gastrointestinal non-neuronal cells including enterocytes and enteroendocrine cells including enterochromaffin cells [44,47]. Specifically, TRPV1 is expressed in the gastrointestinal tract, mainly by primary afferent sensory neurons and enteric neurons and also by mucosal epithelial cells and enteroendocrine cells [48,49]. TRPA1 channels are also expressed in ganglia and enteric primary neurons, which protrude to the gut, and in mucosal cells [45]. Activation of these TRPA1- and TRPV1-expressing neurons release neurotransmitters that may lead to alterations in vascular, immune and smooth muscle functions in the intestine [50]. Both TRPV1 and TRPA1 channels are known to interact with each other in controlling the sensitization dorsal root ganglion neurons [51]; however, whether TRPA1 causes over-expression of TRPV1 or vice-versa remains unknown. At a cellular level, TRP channel proteins perform several functional roles in the gastrointestinal tract. These primarily include their role as molecular sensors and transductors of chemical and physical stimuli, and as effectors of ion channels and receptors, modulating the transport of cations across the plasma membrane [52]. TRP proteins are involved in various cellular functions due to their varied permeability to cations and their ability to influence intracellular Ca^2+^ and Mg^2+^ signaling [53]. In this context, the different microbiome signatures in these KO-mice might be an indirect effect, probably via alterations in Ca^2+^ or Mg^2+^ signals. It has been proposed that the TRP proteins are Ca^2+^ channel/transporters [54]. The Ca^2+^ homeostasis is maintained by efficient response mechanisms involving parathyroid glands, bone, the kidney as well as the intestine [55]. TRPV5 and V6 proteins have been found to act as the gatekeepers of active Ca^2+^ and Mg^2+^ absorption processes in the intestine [53,55], and dysregulation of this influx pathway has been found to be correlated with intestinal malabsorption of Ca^2+^ and Mg^2+^ [56]. However, whether and how TRPA1 and V1 proteins are linked with these signaling influxes remains unknown and requires further investigation. In addition to their contributing role in the absorption of Ca^2+^ and Mg^2+^, TRP channels play a role in secretory mechanisms of the gastrointestinal tract and modulate the alimentary canal motility, contributing to the control of membrane potential and excitability of neurons and epithelial cells [57]. TRPA1, TRPV1 and several other TRP channels are also known to recognize and transduce signals coming from metabolites and environmental toxins, thereby playing an important role in the intrinsic control of a variety of gastrointestinal functions [58]. In addition, TRP channels are involved in the maintenance of blood flow, motor activity, secretory processes and mucosal homeostasis in the gastrointestinal tract and can also influence the function of immune cells such as CD4+ T-cells [45,59]. TRPV1 has also been found to play a role in the intestinal inflammation, pain and hyperalgesia [60]. TRPV1 is upregulated and sensitized during inflammation episodes and its mucosal expression has been found to be linked with pain severity related to functional disorders including irritable bowel syndrome, and quiescent ulcerative colitis and Crohn’s disease [60]. Similarly, TRPA1 has also been found to be upregulated in the intestine of mouse models and human subjects with colitis, ulcerative colitis and Crohn’s disease [61]. The TRPA1 activation on visceral sensory neurons has been shown to stimulate specific neuropeptides, which leads to vasodilatation, local inflammation and mechanical hyperalgesia in the gastrointestinal tract [62]; however, different studies have attributed both pro- and anti-inflammatory effects to TRPA1. Notably, TRPA1 and TRPV1 have also been implicated in the maintenance of immune homeostasis in the gut plausibly via interaction with the gut microbiome. Hence, particularly considering the differences between the distinct intestinal compartments (small and large intestine), further studies are needed to elucidate the precise role of TRP channels in the pathophysiology of gastrointestinal inflammation and diseases. Moreover, further studies using specific Cre-mediated knockout models will be able to decipher if these microbiome differences are exclusively due to gut-specific or systemic KO of TRPs.

The results reported here are, to the best of our knowledge, the first to demonstrate gut microbiome differences in TRPA1 and V1 knockout as well as A1/V1 dKO mice in comparison to their wild-type counterparts. Some of the bacterial taxa reported here have previously been known to be involved in several host metabolic pathways whose association with the neuronal channels involved in pain might be biologically plausible. Moreover, there appears to be a somewhat quantitative association between the abundance of several taxa and the type of TRP channel knockout, hinting at the potential connection between TRP channel proteins and the gut microbiome. Pertaining to the limitations of the present study, it may be noted that our microbiome compositional and functional data are limited to the results obtained from the high-throughput sequencing of the bacterial 16S rRNA gene only and thus the data of microbiome composition and predicted functions should be interpreted conservatively. Further studies employing the whole bacterial metagenomic sequencing approach and also the targeted and untargeted metabolomics analysis of gut microbial metabolites would not only be able to identify more and novel microbial and metabolic biomarkers but may also upgrade the characterization of potentially implicated biological mechanisms and pathways underlying the pain pathophysiology. In addition, further studies employing germ-free models, microbiota transplant strategy, and specific Cre-mediated KO models will be important to examine and validate the casual versus causal implication of gut microbes in pain perception and thermoregulation. It should also be noted that the findings reported in here are from well-established but still rodent models. Hence, it is difficult to be certain whether and how the detected microbiome differences in these TRP KO mice would extrapolate or translate to the human milieus. Furthermore, human subjects suffering from impaired pain perception or pain-related disorders are also highly likely to have a different dietary and lifestyle routines and undertake different therapeutic regimens, all of which could influence their gut microbiota assemblage. Further and more comprehensive studies examining the possible microbiome changes in other similar genotypes and phenotypes of pain and exploring the potential causal versus casual association between the gut microbiome and pain physiology should endeavor to validate and further comprehend this otherwise under-explored microbiome–pain axis. Nevertheless, the data offer avenues to broaden our understanding of the pathophysiology of pain and perhaps could also facilitate outlining the future diagnostic and therapeutic modalities. Exploration of mechanisms by which the gut microbiome and microbial metabolites may affect the functions of TRP receptors could offer novel insights into the pathophysiology of pain and might also possibly lead to the use of microbiome data in the diagnosis of pain in the future. Indeed, if a causal relationship between the gut microbiome and the pathophysiology of pain is established, the way may be paved for the development of novel diagnostic and treatment strategies exploiting this intriguing community of gut microbes. Hence, our data should help to shed some new light on the pathophysiology of pain in the particular context of the intestinal microbial ecosystem and may help reveal novel markers within a biological and physiological framework while improving our understanding of this relatively unknown phenomena of TRP-related pain sensation.

## 4. Materials and Methods

*Animals*. All experiments and procedures were performed in accordance with the North Carolina State University laboratory animal care. The KO mice were generated as per the methods detailed elsewhere [63,64]. Briefly, the KO mice were back-crossed with C57BL6 background for several generations and then KO and control littermates were crossed separately to obtain WT littermates and KO mice. All the genotypes were maintained in similar environment conditions. The mice (male; *n* = 7 per group) were 10–12 weeks old at the time of mechanical pain measurements and the fecal collection. Validation of knockout was assessed by measuring mechanical pain using a von-Frey apparatus (Ugo Basile, Trappe, PA, USA), as described in our previous study [65].

*Gut microbiome analysis*. Gut microbiome was examined as per our previously described methods [66,67,68,69,70]. Briefly, bacterial genomic DNA from fecal specimens was extracted by using a Qiagen DNA Stool Mini Kit (Qiagen, Valencia, CA, USA) with a slight modification described previously [66]. The V4 hypervariable region of the 16S rDNA gene was PCR amplified using the universal primers 515F (barcoded) and 806R; the resulting amplicons were cleaned up with AMPure^®^ magnetic purification beads (Beckman Coulter, Indianapolis, IN, USA); the purified products were quantified using the Qubit-3 fluorimeter (InVitrogen, Carlsbad, CA, USA) and the amplicon library was generated according to methods described elsewhere [71]. The purified PCR product was pooled in equal molar concentrations and sequenced on an Illumina MiSeq platform using 2 × 300 bp reagent kit (MiSeq reagent kit v3; Illumina Inc., San Diego, CA, USA) for paired-end sequencing. The obtained sequences generated were de-multiplexed, quality-filtered, clustered, and taxonomically assigned against Greengenes database with RDP-classifier using the Quantitative Insights into Microbial Ecology (QIIME; http://qiime.org/) software package [72] as described previously [67,68,69]. To avoid bias due to different sequencing depths, the sequences were rarefied to the lowest number of sequences per sample for downstream analyses. Alpha-diversity indices were computed within QIIME. Beta diversity was analyzed using principal coordinate analysis (PCoA) of the unweighted and weighted Unifrac distance (using EMPeror version 0.9.3-dev, https://biocore.github.io/emperor/). Bacterial taxonomy assignment was calculated within QIIME using the default settings to compare the bacterial diversity and abundance between the different groups. The proportions of microbial organism-level phenotypes were computed using the open-source algorithm BugBase. The metabolic and other functional activities were computed using the open source bioinformatics tool Phylogenetic Investigation of Communities by Reconstruction of Unobserved States (PICRUSt; http://picrust.github.io/picrust/) [73]. The sequences were uploaded to PICRUSt and were analyzed for the prediction of functional genes of the classified members of the gut microbiota against Greengenes database. Subsequently, the inferred gene families were annotated against Kyoto encyclopedia of genes and genomes (KEGG) orthologs (Kos) and then collapsed into KEGG pathways to generate the functional pathway. The functions were finally categorized and compared at levels 2 and 3 as per the methods described elsewhere [73].

*Statistical analyses*. α-diversity indices and bacterial abundance between TRP wild-type and KO mice were compared using Kruskal–Wallis test followed by Dunn’s post-hoc multiple pairwise comparison test. Hierarchical clustering and heat-maps depicting the patterns of abundance and log values were constructed R statistical software package (version 3.6.0; https://www.r-project.org/) using the ‘heatmap.2′ and “ggplots” packages. Linear discriminatory analysis (LDA) Effect Size (LEfSe; https://galaxyproject.org/learn/visualization/custom/lefse/) [74] was used to identify discriminative features (unique bacterial taxa) that drive differences in KO versus WT mice. Differences in β-diversity were tested by permutational multivariate analysis of variance, a permutation-based multivariate analysis of variance to a matrix of pairwise distance to partition the inter-group and intra-group distance. Correlation between bacterial abundance and the index of pain measurement was estimated by Spearman’s rank correlation coefficient test (GraphPad Prism software system, version 6.0; https://www.graphpad.com/scientific-software/prism/). In all experiments, significance threshold was set at *p* < 0.05. Unless otherwise stated, all the values presented herein are means ± standard error of mean (SEM).

## Supplementary Materials

The following are available online at https://www.mdpi.com/2076-0817/9/9/753/s1, Figure S1: Hierarchical clustering depicting the closeness of mouse models of TRPA1 knockout (A1KO), TRPV1 knockout (V1KO) and TRPA1V1 double-knockout (A1V1dKO) and TRP wild-type (TRP-WT) to each other on the basis of Euclidean distance between their gut microbiome beta-diversity, Figure S2: Proportions of gut microbiome phenotypes in the mouse models of TRPA1 knockout, TRPV1 knockout and TRPA1V1 double-knockout versus TRP wild-type (TRP-WT) counterparts, Figure S3: LEfSe plot showing the predicted gut microbial metagenomic functions and pathways that are unique in mouse models of TRPA1 knockout (A1KO), TRPV1 knockout (V1KO) and TRPA1V1 double-knockout (A1V1dKO) as well as in TRP wild-type (TRP-WT) counterparts.
