# Supplementary material for: Role of TRP Channels in Shaping the Gut Microbiome
Source: Pathogens. 2020 Sep 16;9(9):753. doi: 10.3390/pathogens9090753 (PMC7559121; doi:10.3390/pathogens9090753)

Suppl. Fig. 1. Hierarchical clustering depicting the closeness of mouse models of TRPA1 knockout (A1KO), TRPV1 knockout (V1KO) and TRPA1V1 double-knockout (A1V1dKO) and TRP wild-type (TRP-WT) to each other on the basis of Euclidean distance between their gut microbiome beta-diversity.

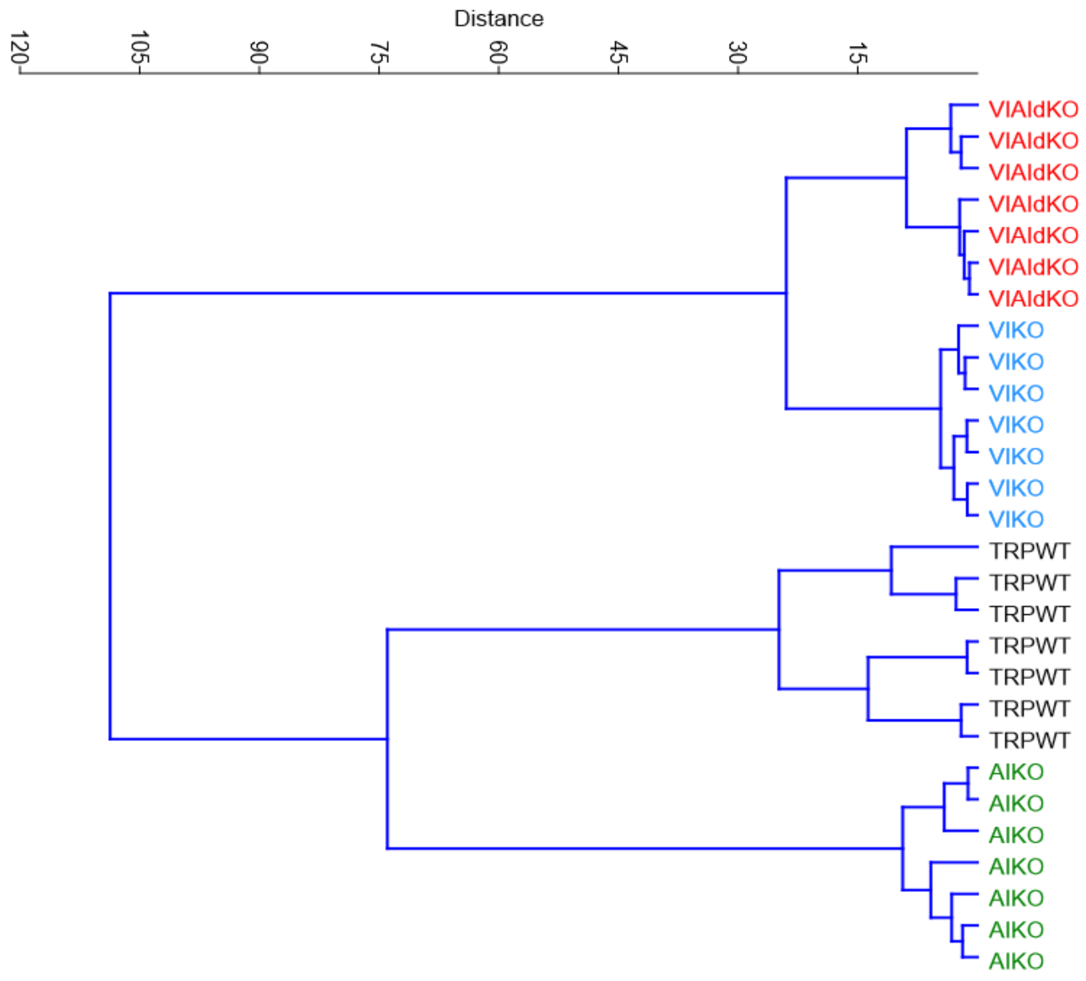

Suppl. Fig. 2. Proportions of gut microbiome phenotypes in the mouse models of TRPA1 knockout, TRPV1 knockout and TRPA1V1 double-knockout versus TRP wild-type (TRP-WT) counterparts.

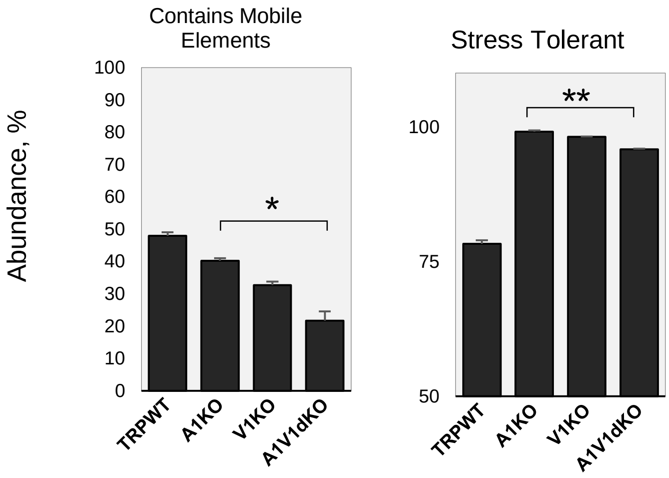

Suppl. Fig. 3. Linear Discriminatory Analysis (LDA) Effect Size (LEfSe) plot showing the predicted gut microbial metagenomic functions and pathways (Level 3 KEGG pathways) that are unique in mouse models of TRPA1 knockout (A1KO), TRPV1 knockout (V1KO) and TRPA1V1 double-knockout (A1V1dKO) as well as in TRP wild-type (TRP-WT) counterparts.

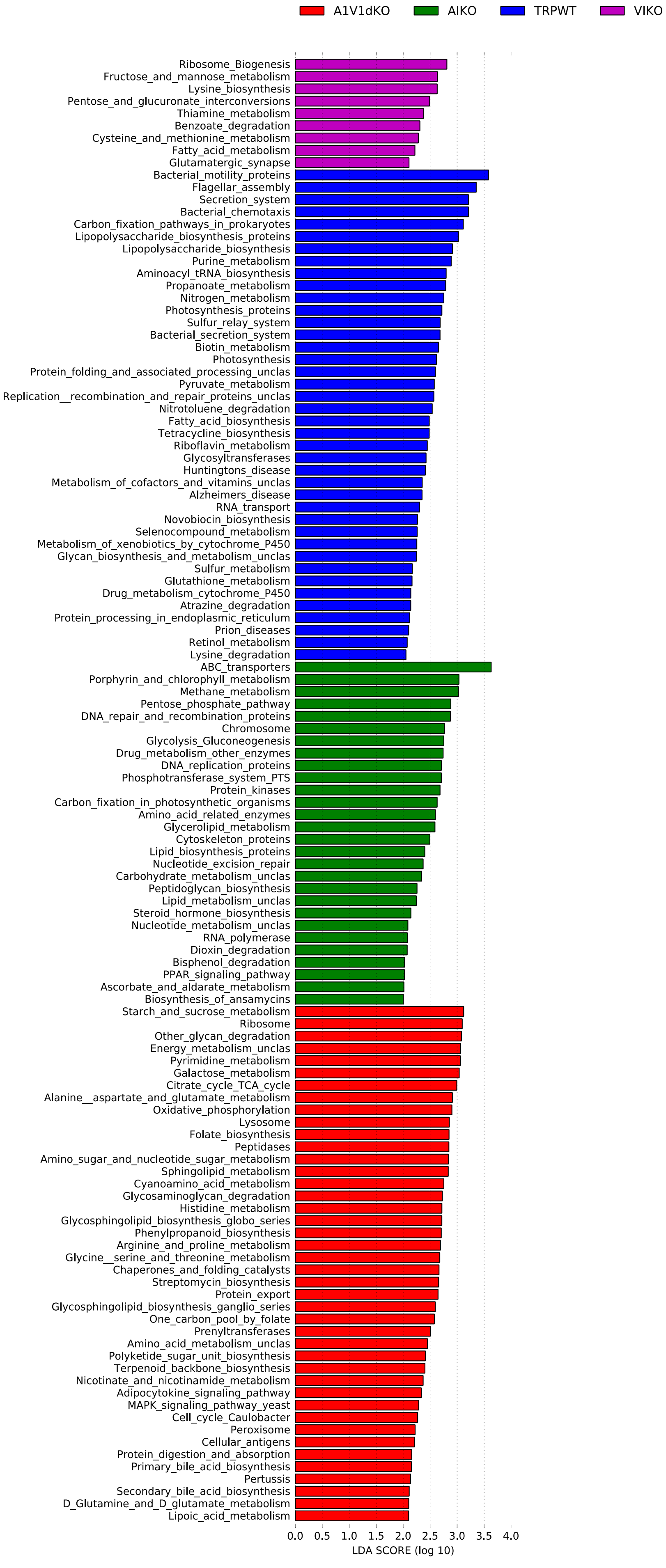

Supplement: Supplementary file 1 [file pathogens-09-00753-s001.pdf]
